# The Association of Serum Biomarkers With Symptomatic Hemorrhagic Transformation in Acute Ischemic Stroke Patients: A Combined Retrospective and Prospective Study

**DOI:** 10.1111/cns.70321

**Published:** 2025-03-26

**Authors:** Shuhua Yuan, Daiquan Gao, Wenjuan Shi, Yue Zhao, Zhengran Guo, Xiaodong Chen, Weili Li, Ke Jian Liu, Jing Yang, Yunzhou Zhang, Xunming Ji, Zhifeng Qi

**Affiliations:** ^1^ Department of Hyperbaric Oxygen Beijing Chaoyang Hospital, Capital Medical University Beijing China; ^2^ Department of Neurology, Cerebrovascular Diseases Research Institute and Clinical Laboratory Xuanwu Hospital of Capital Medical University Beijing China; ^3^ Department of Neurology The First Affiliated Hospital of Shandong First Medical University, Shandong Provincial Qianfoshan Hospital Jinan China; ^4^ Department of Pathology Renaissance School of Medicine, Stony Brook University Stony Brook New York USA

**Keywords:** biomarker, blood–brain barrier (BBB), ischemic stroke, symptomatic hemorrhagic transformation (s‐HT), von Willebrand factor (vWF)

## Abstract

**Background and Purpose:**

Symptomatic intracranial hemorrhage transformation (s‐HT) is a serious complication of ischemic stroke, leading to early neurological deterioration and poor prognosis. It is an urgent problem to timely and effectively identify high‐risk patients with s‐HT at the early stage of stroke. However, so far, there are no effective clinical detection methods or measures. Therefore, the present study aimed to explore novel blood biomarkers related to s‐HT.

**Methods:**

This study includes two parts: a retrospective study and a prospective cohort study. In the first part, s‐HT patients were screened (*n* = 18), and non‐s‐HTs (*n* = 128) were selected from the same period of case patients in the retrospective study cohort. The baseline blood samples were obtained within 30 min of admission, and the levels of 92 proteins related to cerebrovascular diseases were detected using the Olink proteomics technology. Multivariate logistic regression and receiver operating characteristic curves were used to analyze the relationship between serum biomarker levels and s‐HT. In the second part, s‐HT patients (*n* = 28) and non‐s‐HTs (*n* = 130) were selected from a prospective study cohort, which met the same criteria for inclusion and exclusion. Enzyme‐linked immunosorbent assay (ELISA) was used to measure the levels of potential biomarker(s) in serum screened from the first part to confirm its/their association(s) with s‐HT.

**Results:**

Olink assay showed that patients with s‐HT had lower von Willebrand factor (vWF) levels and higher osteoprotegerin, phospholipase C, human insulin‐like growth factor binding protein‐7, matrix metalloproteinase‐2, galectin‐4, spondin‐1 than non‐s‐HTs (*n* = 128) (*p* < 0.005) in a retrospective study cohort. Principal component (PC) and factor analysis showed that the seven biomarkers could explain 62.76% of the variance in the Olink biomarker set, and vWF was a main loading factor in PC2. Multivariate regression analysis showed that a low level of vWF was an independent risk factor (*p* < 0.05) for s‐HT after adjusting for potential confounders. ELISA test results showed that s‐HT patients had a significantly lower vWF levels than the non‐s‐HT group (26.57 [13.64–37.18] vs. 42.00 [26.02–55.52] ng/mL, *p* < 0.001) in the prospective study cohort. Incorporating vWF into the clinical risk factors significantly improved the accuracy of predicting s‐HT (area under the curve [AUC, 0.731 vs. 0.641, *p* < 0.001], [AUC, 0.747 vs. 0.560, *p* < 0.001]) compared to a model employing only clinical risk factors in both study cohorts.

**Conclusion:**

Through the use of a combined retrospective and prospective study, vWF might be a novel blood biomarker for predicting s‐HT occurrence in ischemic stroke patients.

## Graphical abstract

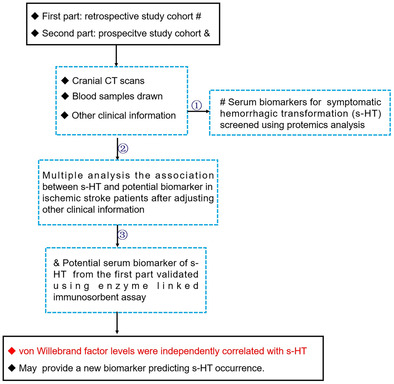

## 1 Introduction

Ischemic stroke is a serious disease that threatens human health worldwide [1]. Symptomatic hemorrhage transformation (s‐HT) is a severe complication of ischemic stroke, which results in early neurological deterioration and poor prognosis [2]. Therefore, it is particularly important to distinguish patients with a high risk of s‐HT at the early stage of stroke.

Multiple clinical risk factors associated with s‐HT have been reported from previous cohort studies, including age, National Institutes of Health Stroke Scale (NIHSS) score, glucose, antiplatelet therapy, and therapy methods, etc. [3, 4, 5]. However, no consensus conclusion on the risk factors associated with s‐HT has been reached, which may be due to sample size, complicated clinical factors, and different treatment measurements, etc. [6]. Therefore, alternative approaches outside routine clinical factors, such as blood biomarkers, may be beneficial.

The mechanism of s‐HT lies in the injury of endothelial cell components in capillaries, which leads to an increase in blood–brain barrier (BBB) permeability [7]. Some studies have shown that serum levels of matrix metalloproteinase‐9 (MMP‐9), cellular fibronectin, and occludin can predict s‐HT in patients with ischemic stroke [8, 9, 10, 11, 12]. To improve the detection of s‐HT, the present study explores new blood biomarkers associated with a higher risk of s‐HT in stroke patients.

In this study, we used Olink proteomics technology to screen significantly different proteins between s‐HT and non‐s‐HT groups from a retrospective study cohort, which were validated using enzyme‐linked immunosorbent assay (ELISA) in a prospective study cohort.

## 2 Methods

### 2.1 Study Design and Participants

This study was conducted in two parts: a retrospective cohort study followed by a prospective cohort study. Both studies were reviewed and approved by the Ethics Committee of Xuanwu Hospital of Capital Medical University, and written informed consent was obtained from all recruited patients or their legally authorized representatives before enrollment.

In the first part, we used data from our previous registration cohort in ischemic stroke collected from December 2021 to December 2022. In the second part, the results of the retrospective study were verified by a prospective cohort established in January 2023 and concluded in January 2024.

Based on the guidelines of the American Heart Association/American Stroke Association for ischemic stroke [13], inclusion and exclusion criteria in both cohorts were as follows: *Inclusion criteria*—(1) diagnosis of ischemic stroke, (2) stroke onset < 24 h, (3) age > 18 years, (4) baseline cranial computed tomography (CT) completed < 30 min of admission, (5) second cranial CT finished within 24–36 h after hospitalization, (6) baseline blood sample obtained within 30 min of admission, and (7) informed consent obtained. *Exclusion criteria*—(1) severe cardiac, liver, and renal dysfunction; (2) coagulation disorder, hemorrhagic disease, and malignant tumor; (3) traumatic stroke and tumor stroke; (4) blood sample hemolysis or cloudy; (5) clinical data incomplete; (6) subarachnoid hemorrhage during following‐up period; (7) bilateral cerebral hemisphere ischemic stroke; and (8) anterior–posterior circulation stroke.

### 2.2 Groups and Subgroups

**FIGURE 1:**
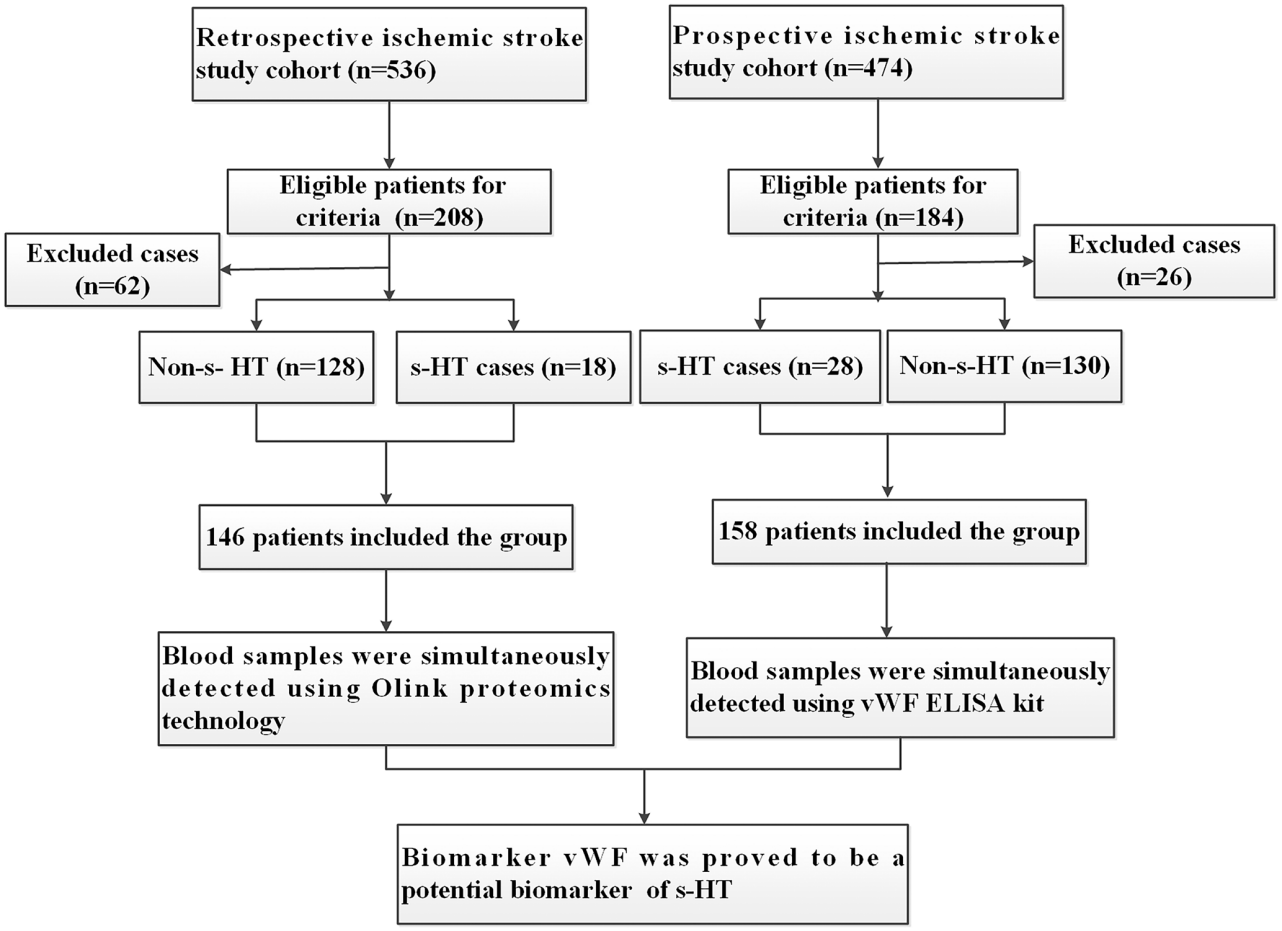
Flowchart of patient selection in both study cohorts. This study was conducted in two parts: a retrospective cohort study followed by a prospective cohort study. ELISA, enzyme‐linked immunosorbent assay; s‐HT, symptomatic hemorrhagic transformation; vWF, von Willebrand factor.

The study patients in both cohorts were divided into two groups, s‐HT and non‐s‐HT, based on the occurrence of s‐HT within 36 h of admission. The final number of patients screened included 146 cases from the retrospective cohort (s‐HT: *n* = 18; non‐s‐HT: *n* = 128) and 158 cases from the prospective cohort (s‐HT: *n* = 28; non‐s‐HT: *n* = 130) (Figure 1).

To further explore other factors associated with the biomarker von Willebrand factor (vWF) in the retrospective cohort, we performed a subgroup analysis. Non‐s‐HT was further divided into non‐HT (*n* = 73) and asymptomatic HT (A‐s‐HT, *n* = 55) subgroups.

### 2.3 Diagnosis of s‐HT

According to the European Cooperative Acute Stroke Study (ECASS) II study [14], diagnostic criteria for s‐HT in the present study are defined as high density in the infarction region on the second cranial CT, accompanied by a baseline NIHSS score increased by more than 4. This assessment was completed by a panel that included clinicians and radiologists. If two doctors of a panel disagreed with the s‐HT diagnosis, a senior doctor would intervene. The investigators participating in the diagnosis of s‐HT were blind to proteomics analysis and vWF detection.

Finally, all the enrolled patients who were not diagnosed with s‐HT were defined as non‐s‐HT, which included non‐HT and A‐s‐HT patients.

### 2.4 Clinical Characteristic Variables

The investigators, who were blind to grouping information, used electronic medical records within 24–72 h of admission to obtain baseline clinical information, including age, sex, previous medical history, clinical measurements, laboratory tests, and therapeutic methods.

The entry of these variables was performed independently by two investigators, and the data were monitored to check consistency.

### 2.5 Blood Samples Obtained

Blood samples were collected within 30 min of admission by the emergency department nurse and then stood at room temperature for 1–2 h. Serum was stored at −80°C as soon as possible after centrifuging.

### 2.6 Olink Proteomics Analysis

The concentration of 92 proteins in the serum samples from the retrospective study cohort was measured using the proximity extension assay technology in Olink proteomics (Shanghai Biotechnology Corporation, China) [15]. Blood samples were randomly distributed on 96‐well plates in a blind way. The protein concentration is expressed on a relative log_2_ scale with arbitrary units presented as normalized protein expression (NPX). More detailed information can be found in Appendix S1.

### 2.7 vWF Levels in Serum Measurement Using ELISA for Study

The levels of vWF in serum from the prospective study cohort were measured using ELISA to verify that its association with s‐HT occurred in acute ischemic stroke. According to the operation manual of the vWF (Lifespan Company, USA), the measurement of vWF levels was completed by a technician who was blind to the grouping information and Olink results.

### 2.8 Model Construction

To determine whether biomarkers improve the ability to distinguish s‐HT, we used multivariate logistic regression analysis to construct different models, including a clinical risk model, protein model, and combined model. The area under the curves (AUC) of the three models was determined using receiver operating characteristic (ROC) curves in both cohorts.

### 2.9 Statistical Analysis

We used SPSS 26.0 software to complete the statistical analysis. All the continuous variables were analyzed using the Kolmogorov–Smirnov test. For normal distribution data, variables are presented as the mean ± standard deviation and compared using independent sample *t*‐tests. For non‐normal distribution, variables are expressed as median (interquartile ranges, IQRs) and analyzed using Mann–Whitney *U* tests. Categorical variables are expressed as frequencies or percentages, which were compared by using chi‐squared tests or Fisher exact tests. *p <* 0.05 was considered a significant difference for all the data.

Adjusted logistic regression was performed on standardized biomarkers using the forced entry method to adjust for the potential risk factors of s‐HT. Principal component analysis and factor analysis were used to analyze the relationship of the study groups. We used multivariate logistic regression and ROC curves to assess the association between biomarkers and s‐HT, and variables with *p* < 0.1 from the univariate regression were selected to enter the multivariate logistic regression analysis in the present study.

The present study is to explore the association between potential biomarker levels and s‐HT in ischemic stroke patients. The sample size was calculated based on a previous study of the median AUC value (0.80) of biomarkers predicting HT and the incidence rate of s‐HT (7%) [10]. The estimated sample size was at least 143 cases. In the present study, there were 146 cases in the retrospective study cohort and 158 cases in the prospective study cohort, which met the sample size requirements.

## 3 Results

### 3.1 Significant Differences in Seven Serum Proteins Were Identified in a Retrospective Study Cohort Using Olink Proteomics Analysis

**FIGURE 2:**
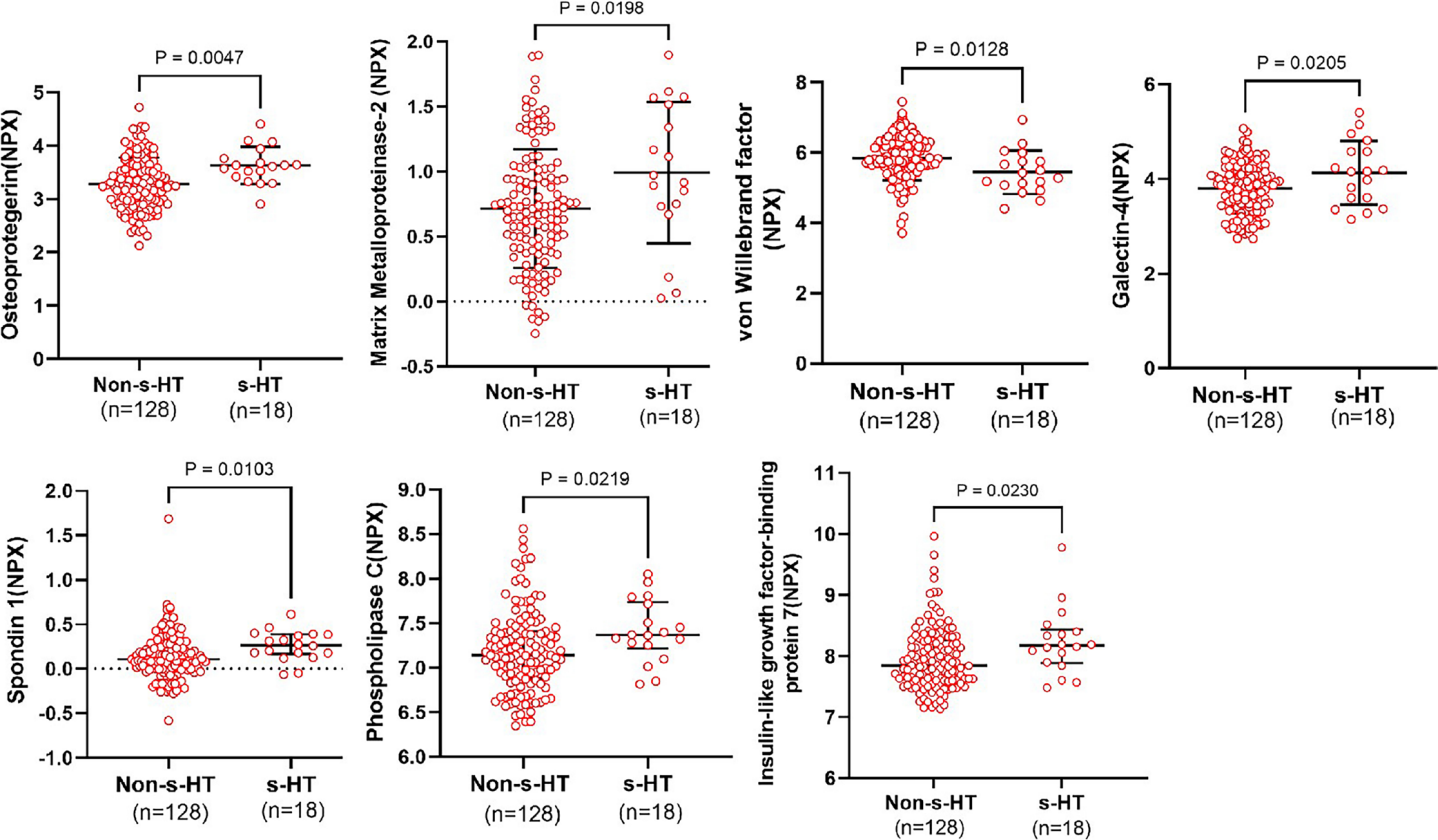
The difference analysis in serum biomarker between study groups in retrospective study cohort using Olink proteomics. Compared with non‐s‐HTs, patients with s‐HT had higher OPG, MMP‐2, Gal‐4, SPON1, PLC, IGFBP‐7 and lower vWF level. The Olink protein levels were displayed as normalized protein expression (NPX), and data were expressed as mean ± SD or median (IQR). Gal‐4, galectin‐4; IGFBP‐7, insulin‐like growth factor binding protein‐7; IQR, interquartile range; MMP‐2, matrix metalloproteinase‐2; OPG, osteoprotegerin; PLC, phospholipase C; SD, standard deviation; SPON1, spondin‐1; vWF, von Willebrand factor.

To identify potential biomarkers associated with s‐HT, we analyzed the difference in the levels of proteins between study groups. Serum samples from the retrospective study cohort were analyzed for potential proteins associated with s‐HT using Olink proteomics, which included 92 biomarkers targeting cerebrovascular diseases III. Seven proteins showed a significant difference (*p* < 0.05, Figure 2) between s‐HT and non‐s‐HT samples. These proteins were osteoprotegerin (OPG), matrix metalloproteinase‐2 (MMP‐2), human insulin‐like growth factor binding protein‐7 (IGFBP‐7), spondin‐1 (SPON1), phospholipase C (PLC), galectin‐4 (Gal‐4), and vWF. The concentration of vWF (NPX) in s‐HT patients was lower than in the non‐s‐HT group (5.44 ± 0.62 vs. 5.83 ± 0.62, *p* = 0.013). However, there were increases in the levels of the six other biomarkers, OPG (3.63 ± 0.35 vs. 3.28 ± 0.49, *p* = 0.005), MMP‐2 (1.00 ± 0.54 vs. 0.71 ± 0.45, *p* = 0.020), IGFBP‐7 (8.17 [7.89–8.43] vs. 7.84 [7.60–8.26], *p* = 0.024), SPON1 (0.27 [0.17–0.39] vs. 0.11 [−0.01 to 0.28], *p* = 0.011), PLC (7.36 [7.22–7.44] vs. 7.14 [6.88–7.41], *p* = 0.023), and Gal‐4 (4.13 ± 0.68 vs. 3.80 ± 0.53, *p* = 0.0205).

### 3.2 Principal Component Analysis of the Retrospective Study Cohort

We conducted a multivariate principal component analysis of the seven proteins to identify the combinations of biomarkers contributing to the best grouping of the dataset.

**FIGURE 3:**
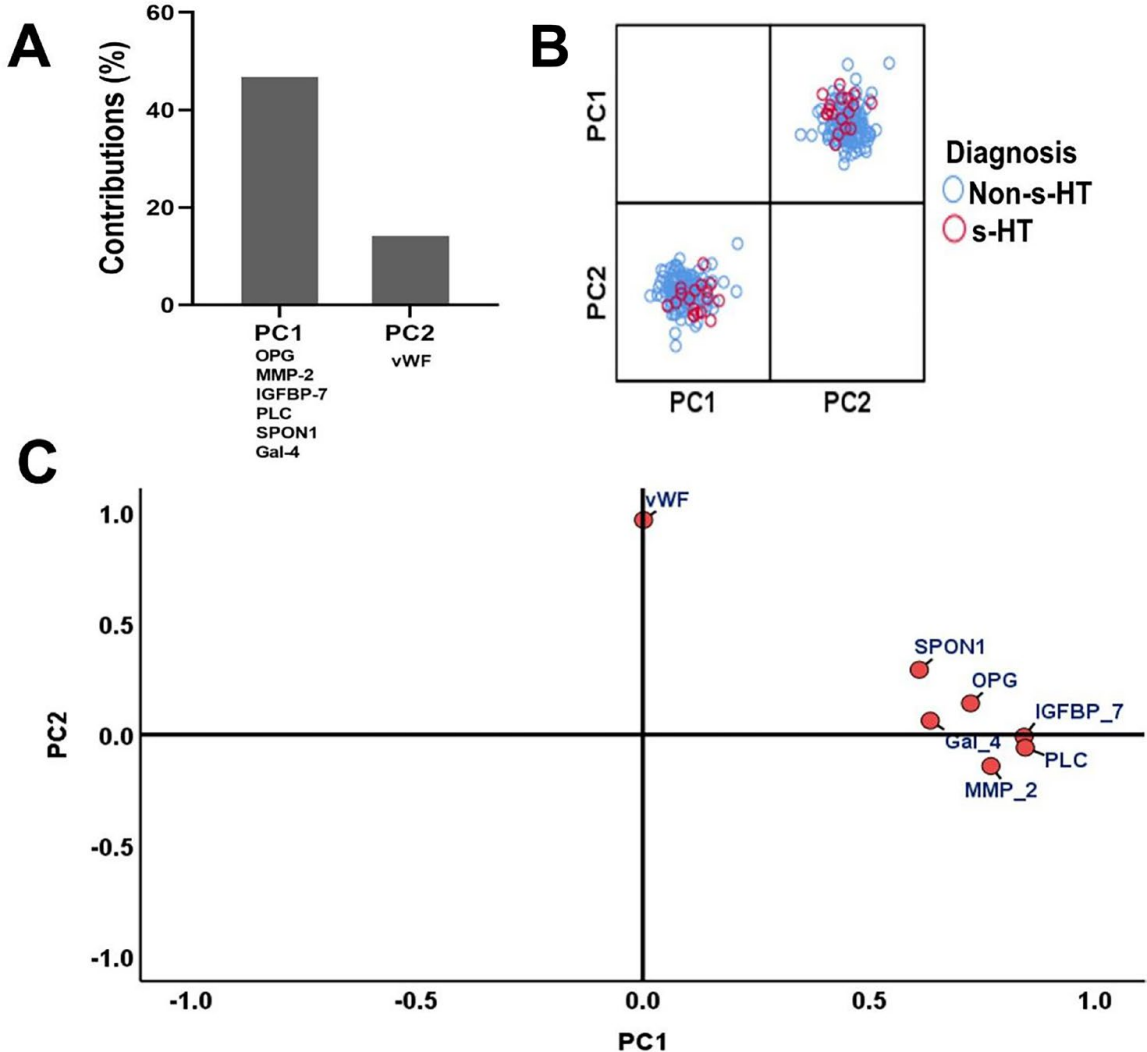
Principal component analysis of Olink biomarkers between study groups. We performed multivariate principal component analysis of seven different Olink biomarkers (*p* < 0.05) between diagnostic groups (non‐s‐HTs and s‐HT patients) to identify combinations of biomarkers contributing to best grouping of the dataset. (A) The relative contribution of different biomarker in each PC. Two main principal components (PC1 and PC2) could explain 62.34% of the variance in the Olink biomarker set. (B) The PC1 and PC2 helped to identify diagnosis category (non‐s‐HTs and s‐HT groups). (C) The loading factors of PC1 included OPG, SPON1, Gal‐4, MMP‐2, PLC, IGFBP‐7, and biomarker vWF was a main loading factor in PC2. Gal‐4, galectin‐4; IGFBP‐7, insulin‐like growth factor binding protein‐7; MMP‐2, matrix metalloproteinase‐2; OPG, osteoprotegerin; PC, principal component; PLC, phospholipase C; s‐HT, symptomatic hemorrhagic; SPON1, spondin‐1; vWF, von Willebrand factor.

We found two main principal components (PC1 and PC2), which could explain 62.76% of the variance in the Olink biomarker set. Figure 3A shows the relative contribution of different biomarkers in each PC. PC1 and PC2 also allowed us to identify the diagnosis categories (non‐s‐HT and s‐HT groups) shown in Figure 3B. From the factor analysis of components, the loading factors of PC1 included OPG, IGFBP‐7, MMP‐2, PLC, SPON1, and Gal‐4, while the biomarker vWF was the main loading factor in PC2 (Figure 3C). These results suggest that the seven biomarkers in PC1 and PC2 could represent the whole spectrum of Olink biomarkers in blood samples. Therefore, it is essential to explore the association between significantly different biomarkers and s‐HT.

### 3.3 Logistic Regression Analysis Indicates That Lower Levels of vWF Are an Independent Risk Factor for s‐HT in the Retrospective Study Cohort

To confirm the potential clinical risk factors of s‐HT, it is necessary to explore baseline characteristic differences between study groups. Compared to non‐s‐HT patients, those with s‐HT had a higher percentage of antithrombotic drug and fibrinogen levels (*p* < 0.05) at baseline. There was no significant difference in other variables between study groups (Table 1). To ensure selected Olink biomarkers are not influenced by clinical factors, it is necessary to adjust for confounding factors.

**TABLE 1:** Main baseline characteristics of the patients included the group according to the presence or absence of symptomatic hemorrhagic transformation in retrospective study cohort.

Baseline characteristics	Total patients (*n* = 146)	Non‐s‐HT group (*n* = 128)	s‐HT group (*n* = 18)	*p*
Male, *n* (%)	105 (71.9%)	95 (74.2%)	10 (55.6%)	0.099
Age (years), mean ± SD	64.27 ± 12.38	64.08 ± 12.79	65.67 ± 9.11	0.612
**Medical history, *n* (%)**				
Hypertension	91 (62.3%)	79 (61.7%)	12 (66.7%)	0.685
Diabetes mellitus	38 (26.0%)	32 (25.0%)	6 (33.3%)	0.451
Dyslipidemia	37 (25.3%)	31 (24.2%)	6 (33.3%)	0.405
Coronary artery disease	24 (16.4%)	19 (14.8%)	5 (27.8%)	0.166
Atrial fibrillation	29 (19.9%)	24 (18.8%)	5 (27.8%)	0.369
Current smoking	72 (49.3%)	65 (50.8%)	7 (38.9%)	0.345
Antithrombotic drug	45 (30.8%)	35 (31.5%)	10 (55.6%)	0.015
Previous stroke	37 (25.3%)	30 (23.4%)	7 (38.9%)	0.158
**Clinical measurement, mean ± SD or median (IQR)**				
Onset to hospital (min)	292.5 (123.3–489.8)	293.50 (125.25–510.25)	283.50 (115.75–355.0)	0.350
Onset to sampling (min)	304.50 (138.25–513.75)	306.00 (139.00–524.50)	289.50 (128.75–435.75)	0.471
Baseline NIHSS score	15 (8.75–20.0)	15.00 (9.00–20.00)	12.50 (6.75–20.25)	0.629
Baseline SBP (mmHg)	151.73 ± 24.29	150.30 ± 24.24	161.83 ± 22.79	0.059
Baseline DBP (mmHg)	82 (73–93)	82.50 (73.00–92.50)	81.5 (76.5–99.25)	0.828
Baseline mRS score	4.00 (4.00–4.00)	4.00 (4.00–4.00)	4.00 (4.00–4.25)	0.714
**Lesion location, *n* (%)**				
Anterior circulation	108 (72.0%)	93 (72.7%)	15 (83.3%)	0.334
Posterior circulation	38 (26.0%)	35 (27.3%)	3 (16.7%)	
**Methods of treatment**				
Nonrecanalization	14 (9.6%)	12 (9.4%)	2 (11.1%)	0.322
Intravenous thrombosis	28 (19.2%)	23 (18.0%)	5 (27.8%)	
Endovascular treatment	73 (50.0%)	66 (51.6%)	7 (38.9%)	
Bridging therapy	31 (21.2%)	27 (21.1%)	4 (22.2%)	
**mTICI score**				
0–1	4 (2.8%)	4	0	0.315
2a	11 (7.6%)	9	2	
2b	31 (21.4%)	30	1	
2c–3	50 (34.5%)	45	5	
**Laboratory findings at admission, median (IQR)**				
WBC (×10^9^/L)	8.77 (6.67–11.04)	8.73 (6.62–11.35)	8.83 (6.67–10.08)	0.600
Glucose (mmol/L)	7.12 (5.93–9.13)	7.11 (5.87–9.14)	7.20 (6.11–8.79)	0.636
Platelets (×10^9^/L)	220.25 ± 58.18	216.53 ± 58.02	217.11 ± 67.70	0.632
APTT (s)	33.65 (31.10–37.13)	33.60 (31.03–36.75)	35.25 (32.95–39.05)	0.253
Fibrinogen (g/L)	3.48 (3.00–4.00)	3.44 (2.97–3.93)	3.80 (3.43–5.05)	0.005
LDL (mmol/L)	2.73 (2.14–3.36)	2.97 (2.17–3.40)	2.49 (1.89–3.21)	0.437

*Note:* The *p* values were calculated using two independent samples *t*‐test, Mann–Whitney *U* test, and chi‐squared test.

Abbreviations: APTT, activated partial thromboplastin time; DBP, diastolic blood pressure; IQR, interquartile range; LDL, low‐density lipoprotein; mRS, modified Rankin Scale; mTICI, modified thrombolysis in cerebral infarction; NIHSS, National Institutes of Health Stroke Scale; SBP, systolic blood pressure; SD, standard deviation; s‐HT, symptomatic hemorrhagic transformation; WBC, white blood cells.

**FIGURE 4:**
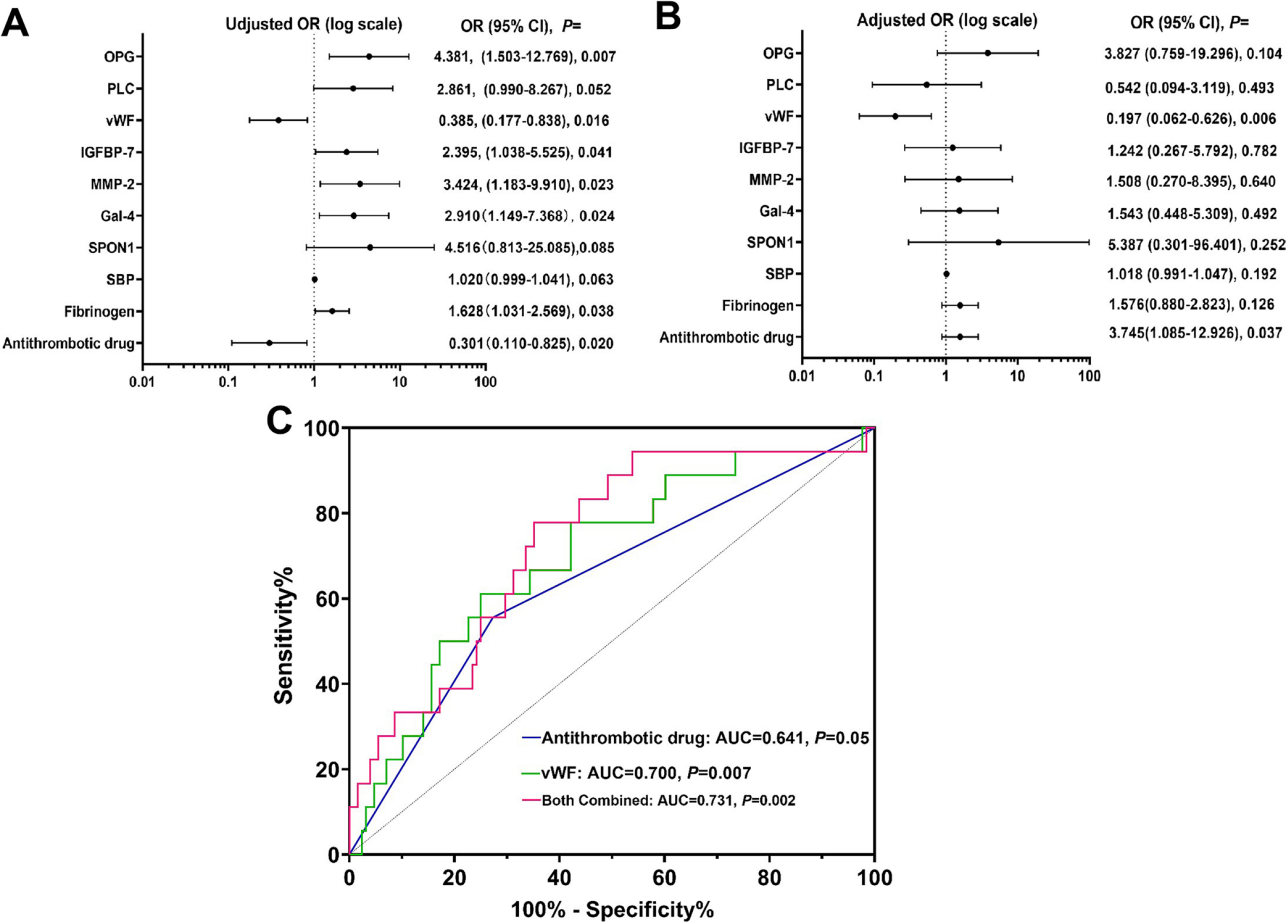
Multivariate logistic analysis of the relationship of Olink biomarkers level and s‐HT in retrospective study cohort. (A) Before adjusting other factors, there are seven potential biomarkers and two clinical risk factors associated with s‐HT, including vWF (OR = 0.385, *p* = 0.016), OPG (OR = 4.387, *p* = 0.007), PLC (OR = 2.861, *p* = 0.052), IGFBP‐7 (OR = 2.395, *p* = 0.041), MMP‐2 (OR = 3.424, *p* = 0.023), Gal‐4 (OR = 2.910, *p* = 0.024), SPON1 (OR = 4.516, *p* = 0.085) and SBP (OR = 1.020, *p* = 0.063), antithrombotic therapy (OR = 3.321, *p* = 0.020), and Fibrinogen (OR = 1.628, *p* = 0.036). (B) After adjusting these potential biomarkers and clinical risk factors, lower vWF (OR = 0.197, *p* = 0.006) remained an independent risk factor of s‐HT in ischemic stroke patients. (C) ROC analysis showed that vWF level had the ability to predict the s‐HT (AUC = 0.700, *p* = 0.007), which was better than clinical index (antithrombotic drug, AUC = 0.641, *p* = 0.05), and could significantly improve the prediction efficiency (AUC = 0.731, *p* = 0.002). The Olink protein levels were displayed as normalized protein expression (NPX), and data were expressed as mean ± SD or median (IQR). Gal‐4, galectin‐4; IGFBP‐7, insulin‐like growth factor binding protein‐7; IQR, interquartile range; MMP‐2, matrix metalloproteinase‐2; OPG, osteoprotegerin; OR, odds ratio; PLC, phospholipase C; ROC, receiver operator characteristic curve; SBP, systolic blood pressure; SD, standard deviation; s‐HT, symptomatic hemorrhagic; SPON1, spondin‐1; vWF, von Willebrand factor.

We conducted a multivariate regression analysis to adjust for confounding factors influencing the association between Olink biomarkers and s‐HT, such as clinical factors. Potential risk factors from univariate regression analysis of *p* < 0.1 were selected and incorporated into multivariate regression analysis, including vWF (odds ratio, OR = 0.385, *p* = 0.016), OPG (OR = 4.387, *p* = 0.007), PLC (OR = 2.861, *p* = 0.052), IGFBP‐7 (OR = 2.395, *p* = 0.041), MMP‐2 (OR = 3.424, *p* = 0.023), Gal‐4 (OR = 2.910, *p* = 0.024), SPON1 (OR = 4.516, *p* = 0.085), SBP (OR = 1.020, *p* = 0.063), antithrombotic therapy (OR = 3.321, *p* = 0.020), and fibrinogen (OR = 1.628, *p* = 0.036) (Figure 4A). After adjusting these potential biomarkers for clinical risk factors, lower vWF (OR = 0.197, *p* = 0.006) remained an independent risk factor for s‐HT in ischemic stroke patients (Figure 4B).

### 3.4 Subgroup Analysis of vWF Level in Acute Ischemic Stroke Patients in the Retrospective Study Cohort

To identify clinical factors that may affect vWF levels, we performed a subgroup analysis. The results showed that patients who experienced a severe stroke had higher vWF levels (*p* < 0.05, Table S1), and a significant positive correlation was observed using multiple linear regression analysis (*β* = 0.267, 95% CI [0.058–0.476], *p* = 0.013) (Table S1).

Interestingly, s‐HT patients had lower vWF levels. This may be associated with the depletion of vWF levels during the onset of ischemia, leading to the development and progression of HT. To confirm this, we stratified the non‐s‐HT group by dividing it into two groups: non‐HT and A‐s‐HT. Compared to non‐HT patients, the A‐s‐HT subgroup had slightly decreased levels of vWF (5.76 ± 0.61 vs. 5.90 ± 0.63, *p* = 0.407), while s‐HT patients had a significant reduction (5.40 ± 0.62 vs. 5.90 ± 0.63, *p* = 0.016) (Table S1, Figure S1). These results further verified the significant association between lower vWF levels and s‐HT.

### 3.5 Olink vWF Biomarker Outperformed Clinical Risk Factors in Distinguishing s‐HT in the Retrospective Study Cohort

To determine whether the vWF can distinguish s‐HT patients from non‐s‐HT, we used ROC analysis. The AUC for distinguishing patients from the non‐s‐HT group was 0.700 (95% CI [0.616–0.770], *p* = 0.004). A cutoff value ≤ 5.50 NPX identified s‐HT patients with a sensitivity of 61.1% and a specificity of 75.0%. Compared to clinical risk factors (antithrombotic therapy, AUC = 0.641, 95% CI [0.558–0.719], *p* = 0.026), the ability of vWF to predict s‐HT was significantly improved (AUC = 0.731, 95% CI [0.651–0.801], *p* = 0.0002) using the Delong test (Figure 4C). These results indicated that the vWF may become a potential biomarker for predicting the occurrence of s‐HT.

### 3.6 vWF Level Measurement Using ELISA in a Prospective Cohort for Study

To validate the power of vWF for predicting s‐HT and improve its clinical applicability, we determined vWF levels in a prospective cohort using ELISA.

The baseline characteristics of patients from the retrospective and prospective cohorts are provided in Table S1. The analysis results showed that there was no statistical difference in many of the variables in both cohorts, including age, sex, previous history, etc. In particular, it is worth mentioning that the two cohorts had a similar percentage of stroke history. This indicated that population characteristics in both cohorts were generally consistent and had good comparability.

**FIGURE 5:**
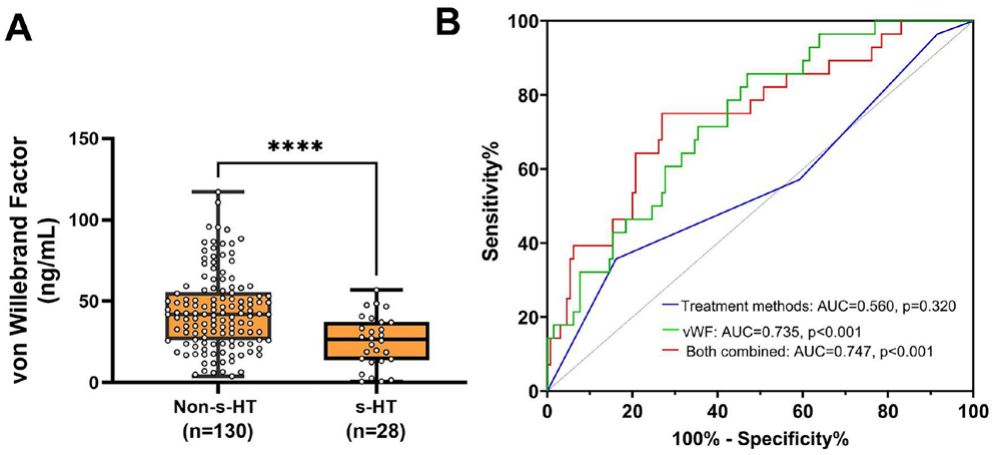
Difference analysis of serum vWF levels between s‐HT patients and non‐s‐HTs in prospective study cohort. (A) Compared to non‐s‐HTs, the serum vWF levels in patients with s‐HT were significantly lower (26.57 [IQR, 13.64–37.18] vs. 42.00 [IQR, 26.02–55.52], *p* < 0.001). (B) The area under the ROC curve of vWF for distinguishing patients with s‐HT from non‐s‐HTs was 0.735 (95% CI [0.642–0.828], *p* < 0.001), which could significantly improve the AUC of clinical index for predicting s‐HT. Data were presented as median (IQR). AUC, area under the curve; CI, confidence interval; IQR, interquartile range; ROC, receiver operator characteristic curve; s‐HT, symptomatic hemorrhagic transformation; vWF, von Willebrand factor.

To guarantee the generalization of the findings, we recruited new stroke patients according to the inclusion and exclusion criteria: non‐s‐HT (*n* = 130) and s‐HT (*n* = 28). Compared with non‐s‐HT patients, those with s‐HT had lower levels of vWF (26.57 [13.64–37.18] vs. 42.00 [26.02–55.52] ng/mL, *p* < 0.001) and could be efficiently distinguished from ischemic stroke patients (AUC = 0.735, *p* < 0.001). A cutoff of vWF at ≤ 40.83 ng/mL can distinguish s‐HT patients with 85.70% sensitivity and 53.10% specificity, as shown in Figure 5A.

### 3.7 vWF Levels Measured Using ELISA Improved the Ability to Predict s‐HT

Compared to non‐s‐HT, we found that therapy methods between study groups were significantly different (Table 2). To screen risk factors associated with s‐HT, we used a multivariate logistic regression analysis. Referred to as nonrecanalization, bridging therapy had a significantly negative correlation with s‐HT (*β* = −1.25, OR = 0.287, 95% CI [0.088–0.935], *p* = 0.038) among different therapy methods. Incorporating the biomarker vWF into the clinical risk factor model (AUC = 0.560, 95% CI [0.479–0.639], *p* = 0.340) significantly improved the prediction of s‐HT (AUC = 0.747, 95% CI [0.672–0.813], *p* < 0.0001) (Figure 5B).

**TABLE 2:** Main baseline characteristics of the patients included the group according to the presence or absence of symptomatic hemorrhagic transformation in prospective study cohort.

Baseline characteristics	Total patients (*n* = 158)	Non‐s‐HT group (*n* = 130)	s‐HT group (*n* = 28)	*p*
Male, *n* (%)	116 (73.4%)	94 (72.3%)	22 (78.6%)	0.496
Age (years), mean ± SD	65.47 ± 12.98	65.02 ± 12.95	67.61 ± 13.16	0.340
**Medical history, *n* (%)**				
Hypertension	107 (67.7%)	90 (69.2%)	17 (60.7%)	0.382
Diabetes mellitus	41 (25.9%)	33 (25.4%)	8 (28.6%)	0.727
Dyslipidemia	33 (20.9%)	28 (21.5%)	5 (17.9%)	0.664
Coronary artery disease	25 (15.8%)	19 (14.6%)	6 (21.4%)	0.370
Atrial fibrillation	33 (20.9%)	27 (20.8%)	6 (21.4%)	0.938
Current smoking	70 (44.3%)	59 (45.4%)	11 (39.3%)	0.556
Antithrombotic drug	39 (24.7%)	32 (24.6%)	7 (25.0%)	0.996
Previous stroke	38 (24.1%)	32 (24.6%)	6 (21.4%)	0.720
**Clinical measurement, mean ± SD or median (IQR)**				
Onset to hospital (min)	270.5 (117.25–270.50)	275.00 (117.25–573.75)	283.50 (115.75–355.0)	0.238
Onset to sampling (min)	284.0 (137.50–521.50)	300.50 (137.50–588.25)	289.50 (128.75–435.75)	0.239
Baseline NIHSS score	12 (6–18)	12.00 (6.00–18.00)	12.50 (6.75–20.25)	0.830
Baseline SBP (mmHg)	155.25 ± 26.72	154.75 ± 26.74	157.54 ± 26.98	0.619
Baseline DBP (mmHg)	84 (73–94)	84.0 (73.00–95.0)	81.5 (76.5–99.25)	0.580
Baseline mRS score	4 (4–5)	4.00 (4.00–5.00)	4.00 (4.00–4.25)	0.835
**Lesion location, *n* (%)**				
Anterior circulation	133 (72.0%)	106 (81.5%)	27 (96.4%)	0.078
Posterior circulation	24 (15.2%)	23 (17.7%)	1 (3.6%)	
**Methods of treatment**				
Nonrecanalization	12 (7.6%)	11 (8.5%)	1 (3.6%)	0.042
Intravenous thrombosis	53 (33.5%)	42 (32.3%)	11 (39.3%)	
Endovascular treatment	62 (39.2%)	56 (43.1%)	6 (21.4%)	
Bridging therapy	31 (19.6%)	21 (16.2%)	10 (35.7%)	
**mTICI score**				
0–1	5 (5.3%)	4 (5.1%)	1 (6.7%)	0.779
2a	11 (11.7%)	10 (12.7%)	1 (6.7%)	
2b	18 (19.1%)	16 (20.3%)	2 (13.3%)	
2c–3	60 (63.8%)	49 (62.0%)	11 (73.3%)	
**Laboratory findings at admission, median (IQR)**				
WBC (×10^9^/L)	8.00 (6.55–9.90)	8.(6.62–11.35)	8.83 (6.67–10.08)	0.839
Glucose (mmol/L)	7.08 (6.05–8.95)	7.11 (5.87–9.14)	7.20 (6.11–8.79)	0.834
Platelets (×10^9^/L)	195.5 (162.75–238.25)	216.53 ± 58.02	217.11 ± 67.70	0.639
APTT (s)	33.75 (31.30–37.03)	33.60 (31.03–36.75)	35.25 (32.95–39.05)	0.783
Fibrinogen (g/L)	3.44 (2.97–4.00)	3.44 (2.97–3.93)	3.80 (3.43–5.05)	0.536
LDL (mmol/L)	2.70 (2.00–3.36)	2.97 (2.17–3.40)	2.49 (1.89–3.21)	0.855

*Note:* The *p* values were calculated using two independent samples *t*‐test, Mann–Whitney *U* test, and chi‐squared test.

Abbreviations: APTT, activated partial thromboplastin time; DBP, diastolic blood pressure; IQR, interquartile range; LDL, low‐density lipoprotein; mRS, modified Rankin Scale; mTICI, modified thrombolysis in cerebral infarction; NIHSS, National Institutes of Health Stroke Scale; SBP, systolic blood pressure; SD, standard deviation; s‐HT, symptomatic hemorrhagic transformation; WBC, white blood cell.

## 4 Discussion

The present study first reported that the lower vWF level tested using Olink proteomics technology was an important risk factor for s‐HT occurrence in a retrospective study cohort, which improved the ability of clinical risk factors to predict s‐HT. Additionally, serum vWF levels in the prospective study cohort were tested using ELISA to demonstrate the conclusion. These results suggested that this study has discovered a novel biomarker associated with s‐HT.

s‐HT is a severe complication of ischemic stroke, especially after recanalization, leading to early neurological deterioration and poor prognosis [16]. Unfortunately, there is currently no method to assess the risk of s‐HT occurrence in patients who have experienced acute ischemic stroke [17, 18, 19]. Based on previous clinical observational studies, a higher NIHSS score, lower ASPECTS (Alberta Stroke Program Early CT Score), massive cerebral infarction, cardiogenic cerebral embolism, and some biomarkers, including MMPs, cellular fibronectin, and occludin, may contribute to s‐HT occurrence for ischemic stroke [9, 20]. However, these indicators are only in an experimental stage and have not been applied to clinical practice. This is mainly because potential biomarkers associated with s‐HT were not selected from many proteins targeting disease and lacked study cohorts to demonstrate reliability as a biomarker. Therefore, the present study design may be innovative and reliable in exploring potential biomarkers of s‐HT occurrence.

Previous studies have also reported the use of proteomics to screen biomarkers by special detection methods that cannot be directly used in the clinic [21, 22]. To improve clinical application and promote the ability of clinical transformation, the ELISA test was used to verify proteins selected using Olink proteomics technology. Therefore, the results of the present study may be more easily and reliably transferred from the bench to the bed compared to the other studies.

It is interesting to find that s‐HT patients in the retrospective study cohort had lower levels of serum vWF, which could efficiently distinguish s‐HT cases from ischemic stroke patients. Moreover, low vWF levels had been demonstrated as an independent risk factor of s‐HT after adjusting for six other biomarkers, which suggests that vWF may be an important biomarker of s‐HT occurrence.

First and foremost, in the present study, we established strict inclusion and exclusion criteria to ensure that patients with congenital vWF deficiencies or other coagulation disorders were not included. Therefore, the patients experiencing s‐HT do not inherently present with low vWF levels prior to the stroke event.

It has been reported that vWF plays an important role in maintaining the balance between bleeding and thrombosis. The vWF is primarily synthesized by endothelial cells [23], and while a small percentage (approximately 6%) exists in a free form, the majority is bound to a range of proteins and structures within the circulation [24]. Before entering the bloodstream, vWF is stored in Weibel–Palade bodies (WPBs) within endothelial cells [25]. Recent studies have reported that blood vWF plays an important role in the development of thrombus formation and atherosclerosis [24, 26]. After microvascular endothelial cell injury, endothelial cells are activated and release the WPBs, including the major component, vWF protein. vWF acts as a molecular bridge connected with platelets and subendothelial collagen to participate in normal hemostasis at sites of vascular injury [27]. Additionally, in a physiological condition, 94% of vWF protein in blood serves as a chaperone to protect FVIII (coagulation factor VIII) from proteolytic inactivation [28]. Therefore, low vWF levels accompanied by low FVIII will aggravate bleeding tendency, which is consistent with our study results.

Our findings indicated a decrease in vWF levels in patients with s‐HT, which aligns with existing literature suggesting that patients with intracranial hemorrhage tend to have lower vWF levels [29]. However, other studies have reported elevated vWF levels in ischemic stroke patients, which are associated with more severe strokes and poorer outcomes at 90 days [30, 31]. The observed discrepancy of vWF levels between bad outcomes and s‐HT after ischemic stroke may relate to differing underlying mechanisms contributing to poor outcomes and BBB damage. It is also important to consider that vWF levels may vary significantly at different time points during the course of a stroke, further complicating the interpretation of these results. Further studies are warranted to investigate the vWF dynamics after ischemic stroke.

Moreover, existing literature indicates that external factors, such as the addition of dipyridamole to aspirin therapy, can reduce vWF expression levels in endothelial cells. This suggests that treatment regimens may influence vWF levels poststroke [32]. Interestingly, our findings demonstrate that the vWF levels in patients with A‐s‐HT and non‐HT patients were higher than those in s‐HT patients. This observation may imply that, in cases of s‐HT, vWF is actively consumed due to interaction with circulating platelets and subendothelial collagen during ischemic events. Consequently, the residual vWF levels may play a critical role in the pathophysiology of s‐HT. Lower vWF levels could diminish platelet adhesion ability, leading to an increased risk of bleeding.

Unlike other studies [33, 34], we used ELISA to test the levels of vWF in newly recruited ischemic stroke patients to assess the clinical transformation ability of vWF using Olink proteomics. The prospective cohort demonstrated the same conclusion as the retrospective cohort, which improved the generalization value of our study results. Moreover, regardless of the study cohort, incorporating vWF into the clinical factor model significantly enhanced diagnostic efficiency. There was no significant difference between vWF alone and the combined model. Therefore, these results demonstrate that the protein model is better than clinical risk factors, which indicates that the vWF might be a potential new blood biomarker of s‐HT from a different perspective.

Our previous research has shown that elevated occludin levels in the bloodstream correlate with increased BBB permeability in animal models of cerebral ischemia and that this elevation is also observed in ischemic stroke patients, which indicated that occludin levels serve as an indicator of BBB integrity and dysfunction. Different from the previous study, serum vWF levels in the present study reflect endothelial cell status and thrombosis risk. So, these studies are essential to advancing our understanding of stroke pathology and improving patient outcomes.

There are some limitations in the present study. Firstly, in our current study, we did not differentiate between the free form and the bound form of vWF. We will explore the roles of free versus bound vWF in future studies to gain deeper insights into their respective impacts on pathological processes such as ischemic injury and hemorrhagic transformation. Secondly, the molecular mechanism of lower vWF levels in s‐HT patients is uncertain and needs to be clarified by an animal study. Thirdly, future studies are needed to determine the changing trend in vWF levels within 36 h of treatment. Finally, due to all blood samples in the present study were collected prior to the initiation of recanalization therapy or any conventional medical treatment, the baseline vWF levels reflect the ischemic injury rather than subsequent reperfusion effects. However, recanalization status undoubtedly influences the outcomes of stroke treatment. Therefore, we plan to expand our cohort to validate the predictive value of vWF levels on admission for s‐HT, along with conducting longitudinal assessments of vWF levels following recanalization therapy.

In summary, we used retrospective and prospective cohort studies to explore the relationship between vWF levels and s‐HT. The vWF level, as an independent risk factor for s‐HT, significantly improves the efficiency of clinical risk factors for the prediction of s‐HT in acute cerebral ischemic stroke. This provides a novel blood biomarker to screen patients with a high risk of s‐HT at early stages of ischemic stroke.

## 5 Conclusion

Through the use of a combined retrospective and prospective study, vWF might be a novel blood biomarker for predicting s‐HT occurrence in ischemic stroke patients.

## Author Contributions

Shuhua Yuan and Daiquan Gao conceived and designed the experiments, performed the experiments, and wrote the paper. Wenjuan Shi, Yue Zhao, Zhengran Guo, and Xiaodong Chen analyzed and interpreted the data. Weili Li, Ke Jian Liu, Jing Yang, and Yunzhou Zhang conceived and designed the experiments. Xunming Ji and Zhifeng Qi conceived and designed the experiments and contributed reagents, materials, analysis tools, or data.

## Conflicts of Interest

Ke Jian Liu is an Editorial Board member of *CNS Neuroscience and Therapeutics* and a co‐author of this article. To minimize bias, he was excluded from all editorial decision‐making related to the acceptance of this article for publication.

## Supporting information


Appendix S1

## Data Availability

Data in the present study are available from the corresponding author on reasonable request.
