# Supplementary material for: The Association of Serum Biomarkers With Symptomatic Hemorrhagic Transformation in Acute Ischemic Stroke Patients: A Combined Retrospective and Prospective Study
Source: CNS Neurosci Ther. 2025 Mar 26;31(3):e70321. doi: 10.1111/cns.70321 (PMC11937931; doi:10.1111/cns.70321)
Supplement: Supplementary file 1 — Appendix S1 [file CNS-31-e70321-s001.docx]

**Supplementary Materials**

**Methods for detailed Olink proteomics analysis**

Serum from the retrospective study cohort were analyzed using the Olink Target 96 at the Shanghai Biotechnology Corporation, Shanghai, China, that had developed the proximity extension assay technology. This panel includes 92 proteins that are either established biomarkers or exploratory proteins with a high potential as disease biomarkers. More detailed information can be found at the Olink website (https://olink.com/products-services/target/biologicalprocess/). The protocols are available online and can be viewed using the link (https://olink.com/content/uploads/2022/05/olink-target-96-short-instructions.pdf). s-HT and non-s-HTs were randomly distributed on the 96 well-plates, and performed in a blind way. The obtained results are reported as cycle threshold (Ct) Normalized Protein expression (NPX) values. NPX values are obtained by normalizing Ct-values against extension non-s-HT, inter plate non-s-HT and a correction factor. NPX values are on log2 scale where a high NPX value corresponds to a high protein concentration and can be linearized by using the formula 2^NPX. Values below the lowest level of detection for each marker, based on the non-s-HTs analyzed in each run, were set to LOD (limit of detection) value.

**Results**

**Table S1. Subgroup analysis the relationship between clinical variables and vWF levels in retrospective study cohort.**

| **Parameters** | **n** | **vWF level (NPX), mean±SD** | ***P* value** |
| --- | --- | --- | --- |
| Age |  |  | 0.510 |
| Total | 146 |  |  |
| ≤40y | 6 | 5.90±0.87 |  |
| 41-60y | 46 | 5.74±0.71 |  |
| ＞60y | 94 | 5.81±0.59 |  |
| Sex |  |  | 0.274 |
| Total | 146 |  |  |
| Male | 105 | 5.75±0.66 |  |
| Female | 41 | 5.88±0.58 |  |
| Hypertension |  |  | 0.958 |
| Total | 146 |  |  |
| Yes | 91 | 5.79±0.65 |  |
| NO | 55 | 5.79±0.61 |  |
| Diabetes |  |  | 0.378 |
| Total | 146 |  |  |
| Yes | 38 | 5.71±0.55 |  |
| NO | 108 | 5.82±0.66 |  |
| Therapy methods |  |  | 0.027**^†^** |
| Total | 146 |  |  |
| No recanalization treatment | 14 | 5.89±0.46 |  |
| Intravenous thrombolysis | 28 | 5.44±0.81 |  |
| Endovascular treatment | 73 | 5.90±0.60 |  |
| Bridge therapy | 31 | 5.79±0.49 |  |
| mTICI score |  |  | 0.300 |
| 0-1 | 4 | 5.59±0.33 |  |
| 2a | 11 | 6.18±0.59 |  |
| 2b | 31 | 5.97±0.59 |  |
| 2c-3 | 50 | 5.80±0.55 |  |
| Onset to sampling |  |  |  |
| Total | 146 |  | 0.266 |
| <270 min | 63 | 5.79±0.68 |  |
| 270-360 | 28 | 5.63±0.76 |  |
| >360 | 55 | 5.87±0.50 |  |
| Baseline NIHSS score |  |  |  |
| Total | 146 |  | 0.006**^§^** |
| Mild stroke (1-4) | 16 | 5.48±0.92 |  |
| Moderate stroke (5-15) | 61 | 5.66±0.52 |  |
| Severe stroke (≥16) | 69 | 5.97±0.60 |  |
| Antithrombotic drug |  |  | 0.312 |
| Total | 146 |  |  |
| Yes | 101 | 5.83±0.67 |  |
| NO | 45 | 5.71±0.56 |  |
| Subgroup of HT |  |  | 0.020^&^ |
| Total | 146 |  |  |
| Non-HT | 73 | 5.90±0.63 |  |
| A-s-HT | 55 | 5.76±0.61 |  |
| s-HT | 18 | 5.40±0.62 |  |

The P values were calculated using the two independent samples t test, and one-way analysis of variance. †: The difference of vWF levels among the four groups was significant (*P*=0.027), while the differences between intravenous thrombolysis and bridge therapy (*P*=0.039), no recanalization treatment and intravenous thrombolysis (*P*=0.030), intravenous thrombolysis and endovascular therapy (*P*=0.001) were still significant by LSD for multiple tests. §: The difference of vWF levels among three groups was significant (*P*=0.006), while the differences between mild stroke and severe stroke (*P*=0.005), moderate stroke and severe stroke (*P*= 0.005) were still significant by LSD for multiple tests. &: The difference of vWF levels among the three groups was significant (*P*=0.02), while the differences between non-HT and s-HT group was still significant by LSD for multiple tests. Abbreviation: vWF, von Willebrand factor; mTICI, modified Thrombolysis in Cerebral Infarction; A-s-HT, asymptomatic hemorrhagic transformation; s-HT, symptomatic hemorrhagic transformation; SD, standard deviation; NIHSS, National Institutes of Health Stroke Scale. LSD, least significant difference. NPX, normalized protein expression.

**Table S2.** **Simple and multiple** **linear regression analysis to evaluate the association of clinical characteristics and vWF levels (NPX) in retrospective study cohort (n=146).**

| **Clinical characteristics** | **Simple linear regression** | | | | **Multiple line regression** | | | | **Multicollinearity** | |
| --- | --- | --- | --- | --- | --- | --- | --- | --- | --- | --- |
|  | **β coefficient 95% CI *P*** | | | | **β coefficient 95% CI *P*** | | | | **Tolerance** | **VIF** |
| **Demographics** |  | | | | | | | | | |
| Age |  | | | | | | | | | |
| 0-40 | 1(reference) | -- | -- | -- | -- | -- | -- | -- | -- | -- |
| 41-60 | -0.158 | -0.706 | 0.389 | 0.568 | -- | -- | -- | -- | -- | -- |
| >60 | -0.086 | -0.618 | 0.445 | 0.749 | -- | -- | -- | -- |  |  |
| Sex |  | | | | | | | | | |
| Male | 1(reference) | -- | -- | **--** |  | -- | -- | -- | -- | -- |
| Female | 0.128 | -0.103 | 0.360 | 0.274 | -- | -- | -- | -- | -- | -- |
| **Medical history** |  | | | | | | | | | |
| Hypertension |  | | | | | | | | | |
| NO | 1(reference) | -- | -- | -- | -- | -- | -- | -- | -- | -- |
| Yes | -0.006 | -0.04 | -0.053 | 0.958 | -- | -- | -- | -- | -- | -- |
| Diabetes mellitus |  |  |  |  |  |  |  |  |  |  |
| NO | 1(reference) | -- | -- | -- | -- | -- | -- | -- | -- | -- |
| Yes | -0.106 | -0.343 | 0.131 | 0.378 | -- | -- | -- | -- | -- | -- |
| Coronary heart disease |  | | | | | | | | | |
| NO | 1(reference) | -- | -- | -- | -- | -- | -- | -- | -- | -- |
| Yes | -0.067 | -0.349 | 0.214 | 0.637 | -- | -- | -- | -- | -- | -- |
| Dyslipidemia |  | | | | | | | | | |
| NO | 1(reference) | -- | -- | -- | -- | -- | -- | -- | -- | -- |
| Yes | -0.075 | -0.315 | 0.164 | 0.535 | -- | -- | -- | -- | -- | -- |
| Current smoking |  | | | | | | | | | |
| NO | 1(reference) | -- | -- | **--** | -- | -- | -- | -- | -- | -- |
| Yes | -0.184 | -0.390 | 0.022 | 0.080 | -- | -- | -- | -- | -- | -- |
| Stroke |  | | | | | | | | | |
| NO | 1(reference) | -- | -- | -- | -- | -- | -- | -- | -- | -- |
| Yes | 0.219 | -0.108 | 0.456 | 0.069 | -- | -- | -- | -- | -- | -- |
| Antithrombotic therapy |  |  |  |  |  |  |  |  |  |  |
| NO | 1(reference) | -- | -- | -- | -- | -- | -- | -- | -- | -- |
| Yes | -0.116 | -0.341 | 0.109 | 0.312 | -- | -- | -- | -- | -- | -- |
| **Therapy methods** |  | | | | | | | | | |
| No recanalization therapy | 1(reference) | -- | -- | -- | 1(reference) | -- | -- | -- | -- | -- |
| Intravenous thrombolysis | -0.444 | -0.844 | -0.044 | **0.030** | -0.312 | -0.577 | 0.047 | **0.021** | 0.896 | 1.115 |
| Endovascular treatment | 0.009 | -0.348 | 0.365 | 0.962 | -- | -- | -- | -- | -- | -- |
| Bridge therapy | -0.109 | -0.502 | 0.285 | 0.586 | -- | -- | -- | -- | -- | -- |
| **Clinical features** |  |  |  |  |  |  |  |  |  |  |
| Baseline NIHSS score |  | | | | | | | | | |
| Mild stroke (1-4) | 1 (reference) | -- | -- | **--** | 1(reference) | -- | -- | -- | -- | -- |
| Moderate stroke (5-15) | 0.181 | -0.160 | 0.521 | 0.296 | -- | -- | -- | -- | -- | -- |
| Severe stroke ≥16 | 0.489 | 0.153 | 0.826 | **0.005** | 0.267 | 0.058 | 0.476 | **0.013** | 0.896 | 1.115 |
| Onset to blood sampling |  | | | | | | | | | |
| <270 min | 1(reference) | -- | -- | -- | -- | -- | -- | -- | -- | -- |
| 271-360 min | -0.164 | -0.449 | 0.120 | 0.256 | -- | -- | -- | -- | -- | -- |
| >360 min | 0.076 | -0.155 | 0.307 | 0.516 | -- | -- | -- | -- | -- | -- |

Abbreviation: NPX, normalized protein expression; VIF, variance inflation factor; CI, confidence interval; NIHSS, National Institute of Health Stroke Scale.

**Table S3. Baseline characteristics comparison in retrospective and prospective study cohorts.**

| **Baseline characteristics** | **Total patients (n=304)** | **Retrospective cohort (n=146)** | **Prospective cohort(n=158)** | ***P* value** |
| --- | --- | --- | --- | --- |
| Male, n (%) | 221(72.7%) | 105 (71.9%) | 116(73.4%) | 0.769 |
| Age (y), mean±SD | 64.9±12.69 | 64.27±12.38 | 65.47±12.98 | 0.501 |
| **Medical history, n (%)** |  |  |  |  |
| Hypertension | 198(65.1%) | 91 (62.3%) | 107（67.7%） | 0.324 |
| Diabetes mellitus | 79(26.0%） | 38 (26.0%) | 41（25.9%） | 0.998 |
| Dyslipidemia | 70(23.0%) | 37(25.3%) | 33(20.9%) | 0.356 |
| Coronary artery disease | 49 (16.1%) | 24(16.4%) | 25(15.8%) | 0.884 |
| Atrial fibrillation | 62(20.4%) | 29(19.9%) | 33(20.9%) | 0.825 |
| Current smoking | 142(46.7%) | 72 (49.3%) | 70(44.3%) | 0.382 |
| Antithrombotic drug | 84(27.6%) | 45 (30.8%) | 39 (24.7%) | 0.232 |
| Previous stroke | 75(24.7%) | 37(25.3%) | 38(24.1%) | 0.794 |
| **Clinical measurement, mean±SD or median (IQR)** |  |  |  | |
| Onset to hospital (min) | 281.5 (119.75-491.25) | 292.50 (125.25-489.75) | 270.50 (117.25-495.75) | 0.933 |
| Onset to sampling (min) | 295(138.25-515.25) | 304.50 (138.25-513.75) | 284.0(137.5-521.5) | 0.864 |
| Baseline NIHSS score | 14.0 (7.00-19.75) | 15.00 (8.75-20.0) | 12.0 (6.00-18.00) | **0.015** |
| Baseline SBP (mmHg) | 153.56 ± 25.60 | 151.73±24.29 | 155.25±26.72 | 0.231 |
| Baseline DBP (mmHg) | 82 (73-93) | 82.50 (73.00-92.50) | 81.5(76.5-99.25) | 0.370 |
| Baseline mRS score | 4.0(4-4.75) | 4.00 (4.00-4.00) | 4.00 (4.00-5.00) | 0.914 |
| **Lesion location, n (%)** |  |  |  | **0.019** |
| Anterior circulation | 242 (79.6%) | 108(74.0%) | 134(84.8%) |  |
| Posterior circulation | 62 (20.4%) | 38(26.0%) | 24(15.2%) |  |
| **Methods of treatment** |  |  |  | **0.040** |
| Non-recanalization | 26(8.6%) | 14 (9.6%) | 12 (7.6%) |  |
| Intravenous thrombosis | 81(26.6%) | 28 (19.2%) | 53 (33.5%) |  |
| Endovascular treatment | 135(44.4%) | 73(50.0%) | 62(39.2%) |  |
| Bridging therapy | 62(20.4%) | 31 (21.2%) | 31 (19.6%) |  |
| **mTICI score** |  |  |  | 0.224 |
| NA | 113 （37.2%） | 50 (34.2%) | 63（39.9%） |  |
| 0-1 | 9 （3.0%） | 4 (2.7%) | 5（3.2%） |  |
| 2a | 22（7.2%） | 11(7.5%) | 11（7.0%） |  |
| 2b | 49 （16.1%） | 31 (21.2%) | 18（11.4%） |  |
| 2c-3 | 111 （36.5%） | 50 (34.2%) | 63（39.9%） |  |
| **Laboratory finding at admission, median (IQR)** |  | | | |
| WBC (×10^9^/L) | 8.31(6.60-10.29) | 8.77(6.67-11.04) | 8.01 (6.55-9.90) | **0.047** |
| Glucose(mmol/L) | 7.09(6.00-9.03) | 7.12(5.93-9.13) | 7.08 (6.05-8.95) | 0.729 |
| Platelets (×10^9^/L) | 205.5(168.00-247.00) | 216(181-255.25) | 195.50(162.75-238.25) | **0.011** |
| APTT(s) | 33.65(31.13-37.10) | 33.60 (31.1-37.13) | 33.75 (31.3-37.03) | 0.253 |
| Fibrinogen (g/L) | 3.46(3.00-4.01) | 3.49 (3.02-4.02) | 3.44 (2.97-4.00) | 0.628 |
| LDL (mmol/L) | 2.72(2.09-3.35) | 2.73 (2.14-3.35) | 2.70 (2.00-3.36) | 0.708 |

The P values were calculated using two independent samples t test, Mann-Whitney U test and chi-square test. Abbreviation: SD, standard deviation; IQR, interquartile range; NIHSS, National Institutes of Health Stroke Scale; SBP, systolic blood pressure; DBP, diastolic blood pressure; mRS, modified Rankin Scale; mTICI, modified Thrombolysis in Cerebral Infarction; WBC, white blood cells; APTT, activated partial thromboplastin time; LDL: low density lipoprotein.

**Subgroup analysis of vWF level between non-s-HT and s-HT patients**

To further explore the vWF level for asymptomatic HT (A-s-HT), non-s-HT group were divided into non-HT and A-s-HT patients. We used the one-way analysis of variance to analyze the vWF difference of study groups. Results showed that s-HT patients had significantly lower levels of vWF and A-s-HT had a slightly reduction compared to non-HT patients.


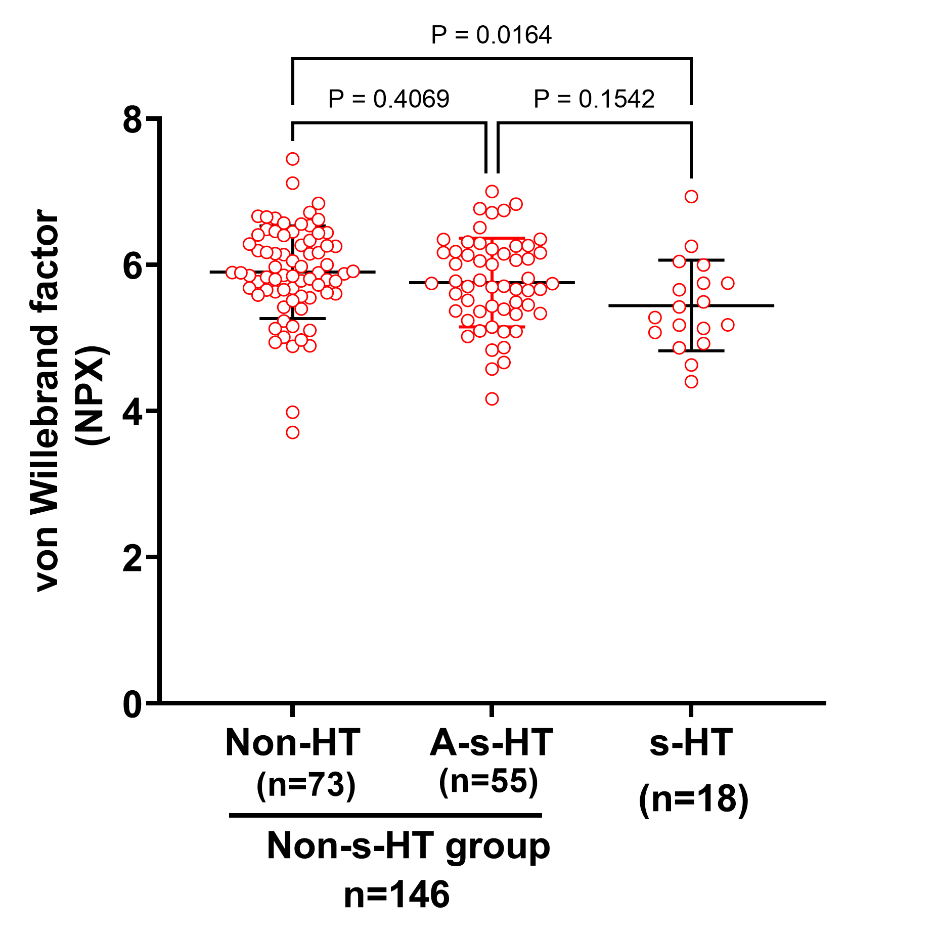


**Figure S1. The difference analysis of vWF levels between non-s-HT subgroup and s-HT group in retrospective study cohort.** Compared to non-HT patients, the vWF levels with A-s-HT in non-s-HT group had a downward tendency (5.90±0.63 vs 5.76±0.61, *P*=0.407); while, the s-HT patients had a significantly lower levels of vWF (5.9±0.63 vs 5.4±0.62, *P*=0.016). Data were expressed as mean±SD. Abbreviation: A-s-HT, asymptomatic hemorrhagic transformation; s-HT, symptomatic hemorrhagic transformation. SD, standard deviation; vWF, von Willebrand factor.
